# Establishment of a novel human lymphoblastic cell strain with the long arm of chromosome 11 aberration without *MLL* rearrangement

**DOI:** 10.1038/s41598-017-00874-6

**Published:** 2017-04-13

**Authors:** Qian Wang, Lin Zhuang, Pei Li, Qiang Niu, Ping Zhu, Miao-Xia He, Hui Jiang, Chang-Cheng Liu, Min-Jun Wang, Li Chen, Hui Cheng, Yan Ma, Xiao-Xia Hu, Yi-Ping Hu, Xiao-Ping Xu

**Affiliations:** 1 Department of Hematology, HuaShan Hospital Affiliated of FuDan University, No. 12 Middle WuLumuqi Road, Shanghai, 200040 P.R. China; 2 Department of Pathology, ChangHai Hospital Affiliated of Second Military Medical University, No. 168 Changhai Road, Shanghai, 200433 P.R. China; 3 Department of Cell Biology, Second Military Medical University, No. 800 Xiangyin Road, Shanghai, 200433 P.R. China; 4 Department of Hematology, ChangHai Hospital Affiliated of Second Military Medical University, No. 168 Changhai Road, Shanghai, 200433 P.R. China

## Abstract

At present, all cell strains derived from acute lymphoblastic leukemia (ALL) patients with the long arm of chromosome 11 aberration are accompanied with mixed lineage leukemia (*MLL*) gene rearrangement. In this study, we established a permanent ALL cell strain CHH-1 with the long arm of chromosome 11 aberration and without *MLL* rearrangement, hoping that it could be used for the research of ALL with such genetic abnormality. CHH-1 cell strain was certified through morphology, immunophenotype, genetics and immunoglobulin (Ig) gene rearrangement analysis. Cell characteristics including tumorigenic ability, semisolid colony forming ability, telomerase activity, autocrine and invasion were further detected. Cells were with an add(11)(q23) structural abnormality without *MLL* rearrangement, and were consistent with the genetic abnormality of the patient. In addition, these cells had features of tumor-forming ability, high colony forming capacity, unique cytokine autocrine mode, high telomerase activity, and high invasion ability. CHH-1 may prove to be a useful cell model for the research of human leukemia with genetic aberration in chromosome 11, and help explore the role of such genetic abnormality in the pathogenesis, progression and prognosis of ALL, and in developing new target drugs.

## Introduction

Acute lymphoblastic leukemia (ALL) is a malignancy that originates from hematopoietic precursors of the lymphoid lineage. A purely leukemic presentation is most typical of B lineage ALL (85%)^1^. It is the most common leukemia in children, which accounts for approximately 80% of all leukemias in this group and 20% in adults. The complete remission (CR) rate of adult ALL ranges from 70% to 90%, with a 5-year overall survival (OS) rate of below 30% due to its high relapse rate^2^.

With the advances in cytogenetic and molecular techniques over the past 20 years, our understanding about the biology and pathogenesis of leukemia has progressed tremendously. Chromosomal abnormalities have become increasingly significant biomarkers in the diagnosis, prognostics, detection of residual disease and targeted therapy of ALL. The normal number of chromosomes with structural abnormalities is the most frequent abnormal karyotype in adult ALL^3, 4^. Structural abnormalities in the long arm of chromosome 11 are frequently found in ALL, and are associated with poor prognosis^5^. Recently, most studies have focused on *MLL* gene rearrangement at 11q23^6^. However, the *MLL* gene is not rearranged in most of other cases, suggesting that these patients may have breakpoints at 11q22-q25 beyond the *MLL* gene. A previous study^7^ analyzed 40 adult leukemia patients with the 11q22-q25 breakpoint without *MLL* rearrangement, and suggested that some genetic loci except *MLL* in this area may be associated with the pathogenesis of leukemia. However, there is little knowledge on the role of such genetic abnormality in ALL. One of the main reasons for this is the lack of corresponding cell lines. It has been acknowledged that continuous human leukemia-lymphoma cell lines are well-sourced, accessible and manipulable living cells that have significantly contributed to the better understanding of the pathophysiology of hematopoietic tumors^8^. However, no continuous human leukemia-lymphoma cell line carry the chromosome 11 abnormality without *MLL* rearrangement^9^. Therefore, very few cell-based experiments on this genetic abnormality have been carried out.

In this study, we established a novel human lymphoblastic cell strain CHH-1 with the long arm of chromosome 11 aberration without *MLL* rearrangement, which was authenticated to be derived from the same B-ALL leukemia clone of the same patient and possess the characteristics of high telomerase activity, a unique growth factor autocrine mode with high invasion ability. This novel permanent and stable B lymphoblastic cell strain may prove to be a useful and distinctive model for the research of human leukemias with this type of chromosome 11 structure aberrations.

## Materials and Methods

### Case report

The CHH-1 cell strain was derived from a 66-year-old Chinese man with ALL. The patient was admitted to Huashan Hospital affiliated to Fudan University (Shanghai, China) in September 2013 for ostalgia and fever. Physical examination on admission revealed sternal tenderness. Laboratory examination revealed: hemoglobin (Hb) 7.2 g/dl, platelet count 28 × 10^9^/L, and white blood cell (WBC) count 1.81 × 10^9^/L. Bone marrow examination revealed hypercellular marrow with 82% blasts, which negative for peroxidase (POX) staining and positive for periodic acid-schiff (PAS) staining. Flow cytometry was positive for CD10, human leukocyte antigen (HLA-DR), CD19, terminal deoxynucleotidyl transferase (TdT), CD79a, CD34, CD20 and CD38, and negative for CD3, myeloperoxidase (MPO), CD5, CD15, CD2, CD4, CD56, CD7, CD117, CD1a, CD13, cytoplasmic IgM (cyIgM), CD11c, CD64, CD138, CD33, CD16, CD4 and CD8; which was defined as the common B subgroup according to the European Group for the Immunological characterization of leukaemias (EGIL) standard. Karyotype analysis of the bone marrow revealed 46, XY, add(11)(q23) [7]/46, XY [13]. The chimaeric messenger RNA (mRNA) screening was negative such as *dupMLL*, *MLL*/*AF4*, *MLL*/*AF6*, *BCR*/*ABL*, *MLL*/*AF1P*, *MLL*/*AFX*, *MLL*/*ENL*, *E2A*/*PBX1*, *TEL*/*AML1*, *SIL*/*TAL1*, *TLS*/*ERG*, *E2A*/*HLF*, *TEL*/*ABL*, and *HOX11*. Following the diagnosis of ALL (Common-B), the patient received chemotherapy including vincristine, darubicin, cyclophosphamide, urbason and pegaspargase. After two months, the blasts in the bone marrow reduced to 1%, achieving CR. However, he relapsed in April 2014 and re-induction chemotherapy did not lead to CR. The patient died from bleeding in May 2014. This study was approved by the institutional committee of Huashan Hospital.

### Ethics Approval and consent to participate

I confirm that I have read the Editorial Policy pages. This study was conducted with approval from the Ethics Committee of our hospital. This study was conducted in accordance with the declaration of Helsinki. Written informed consent was obtained from all participants.

### Cytokines

All cytokines including recombinant human stem cell factor (rhSCF), interleukin-3 (rhIL-3), rhIL-2, rhIL-10, rhIL-22, leukemia inhibitory factor (rhLIF), tumor necrosis factor-α (rhTNF-α) and fms-like tyrosine kinase ligant (rhFlt3lig) used in this study were provided by Pepro Tech Co., Ltd. (Rocky Hill City, USA).

### Cell culture and cloning

Primary bone marrow cells were obtained from the patient at first diagnosis of ALL in September 2013. The patient provided an informed consent. Mononuclear cells were isolated and separated by Ficoll-Hypaque and first seeded in culture medium on September 9, 2013. The primary cells were cultured in a 24-well plate (Corning, NY, USA) at a density of 2 × 10^6^/ml in RPMI-1640 medium (Gibco, Grand Island, NY, USA) containing 20% heat-inactivated fetal bovine serum (FBS; Gibco, Grand Island, NY, USA) and 50 ng/ml of both rhIL-3 and rhSCF at 37 °C with a 5% carbon dioxide (CO_2_) atmosphere. The medium was replaced every 3–4 days depending on the cell growth rate. Cells were examined daily under an inverted microscope, and the cell number was counted weekly in a standard hematocytometer using trypan blue dye exclusion. Fresh medium was added regularly to keep the cells at a density of 2 × 10^6^/ml. After 11 weeks, the cell number increased by 2–3 fold, and recombinant cytokines were omitted from the culture medium after 12 weeks. Cells were transferred into a 96-well plate (Corning, NY, USA) for monoclonal culture by limiting dilution assay on January 3, 2014. Then, the monoclonal amplified cells were transferred to flasks (Corning, NY, USA) for further continuous culture after four weeks in RPMI-1640 medium containing 10% FBS. In this study, additional B lymphoblastic or lymphoma cell lines MC116, Raji and Toledo were also used and cultured in RPMI-1640 medium containing 10% FBS.

### Cell morphologic and cytochemical assay

Morpholigical characteristics of living cultured cells were observed under an inverted microscope (Olympus, Tokyo). Cytocentrifuged smears were stained with Wright, POX, PAS, nonspecific esterase (NSE) and sodium fluoride (NaF) inhibited reaction. Cell morphology was observed by heavy metal staining under an electron microscope.

### Immunophenotyping and cell sorting

For the detection of the immunophenotype of the patient sample and CHH-1 cell strain, the following antibodies were used: PE anti-MPO, HLA-DR, CD19, CD20, CD79α, CD23, CD38, CD138, CD5, CD33, CD117, CD56, CD11c, λ, FITC anti-CD3, CD34, TdT, CD10, IgM, CD22, CD64, CD7, CD13, CD14, CD15, k, and PerCP anti-CD45 (Becton Dickinson Inc., USA). Positivity for the antigens was determined using a FACSCalibur flow cytometer (Becton Dickinson Inc., USA). Using CD19 conjugated magnetic microbeads (Miltenyi Biotec, Auburn, CA), CD19 positive cells were isolated from peripheral blood mononuclear cells (PBMCs) or bone marrow mononuclear cells (BMMNCs), according to manufacturer’s instruction.

### Cytogenetic and Ig gene rearrangement analysis

Cytogenetic study was performed on the cell strain by culturing cells in 10% FBS RPMI-1640 medium for 48 hours, and treating them with colcemid. Chromosomes were stained using the conventional quinacrine mustard banding technique. The karyotype was determined according to the International System for Human Cytogenetic Nomenclature (ISCN) criteria. Fluorescence *in situ* hybridization (FISH) was performed according to manufacturer’s protocols using the *MLL* dual-color break-apart probe (Vysis, Bergisch Gladbach, Germany). Images were captured using a charge coupled device (CCD) camera configured to a fluorescence microscope (Zeiss, Gottingen, Germany) and analyzed using monochromatic special software (Quips, Applied imaging, Newcastle, UK).

Genomic DNAs were isolated from cells using a Maxwell RSC Cultured Cells DNA Kit (Promega), according to manufacturer’s instructions. The DNAs were analyzed using multilocus primers designed for IgVH-A (FR1-JH), IgVH-B (FR2-JH), IgVH-C (FR3-JH), IgDH-A (DH1-6-JH), IgDH-B (DH7-JH), Igκ (Vκ-Jκ) and Igλ (Vλ-Jλ) (Simplegen Corporation, Shanghai, China). The polymerase chain reaction (PCR) system including the GoTaq GreenMaster Mix (Promega), primers mix and genomic DNA was reacted at 95 °C for 15 minutes and 4 °C for 60 minutes. PCR products were visualized in polyacrylamide gels stained with SYBR Green I (Invitrogen).

### Proliferation study and the effect of cytokines on CHH-1 cell proliferation

CHH-1 cell doubling time was calculated as the equation: T_D_ = t lg 2/lg(Nt/N0). Cell proliferation and cell death were assessed using trypan blue staining. Cells were cultured in a 24-well round-bottom plastic culture plate (Corning, NY, USA) at 1 × 10^4^ cells per well in RPMI-1640 medium with 10% FBS, and grown for six days. Cells in every three wells were separately enumerated for the number of viable cells each day. Cells in each well were enumerated in triplicates, and the mean number of three wells was recognized as the daily mean value.

The cell cycle was analyzed using the propidium iodide (PI) fluorescent staining method. Log-growth cells were harvested (1 × 10^6^) and fixed in precooled 70% ethanol at −20 °C overnight. Then, cells were collected by centrifugation and washed with phosphate buffered saline (PBS). Subsequently, cells were incubated with RNaseA at 37 °C for 30 minutes and with PI at 4 °C for 30 minutes in the dark. DNA content was analyzed on a FACSCalibur flow cytometer, and data were analyzed using the Modfit LT 3.2 software (Verity Software House, ME, USA).

The proliferative response of CHH-1 cells to cytokines rhIL-2 (50 ng/ml), rhIL-10 (100 ng/ml), rhIL-22 (100 ng/ml), rhLIF (100 ng/ml), rhTNF-α(50 ng/ml) and rhFlt3lig (100 ng/ml) was examined by carboxyfluorescein diacetate succinimidyl ester (CFDA-SE) Cell Proliferation Assay (Beyotime). Cells were stained with CFDA SE according to manufacturer’s instructions, and cultured in 6-well plates with various cytokines or in the absence of cytokines for 72 hours. CFDA SE dilution was analyzed by flow cytometry on a FACSCalibur, and data were analyzed using the FlowJo software (Treestar, Ashland, OR, USA). Each cytokine group and control group were analyzed in triplicates.

### Clonal growth in semisolid culture

CHH-1 cells or normal CD19 + PBMCs were plated at 2 × 10^2^ cells per well in 1% semi-solid methylecellulose (Difco Laboratores, Detroit, Mich., USA) containing Iscove’s Modified Dulbecco’s Minimal Essential Medium (IMDM; Gibco, Grand Island, NY, USA) with 30% FBS. As a growth factor, 10 ng/ml of rhIL-3, 50 ng/ml of rhSCF and 10 ng/ml of granulocyte-macrophage colony stimulating factor (rhGM-CSF) were added. Then, cells were incubated for 14 days at 37 °C with 5% CO_2_ in a humidified environment. Colonies with more than 50 cells were scored using a dissecting microscope. Data were represented as means ± standard deviation (SD) of triplicate samples.

### Tumorigenicity in NOD/SCID mice

Cultured 5 × 10^6^ CHH-1 cells were subcutaneously injected into the right armpit of 6-week-old male NOD/SCID mice, and mice injected with 0.2 ml of PBS in the left armpit was used as control (*n* = 12). At designated intervals, tumor size was measured as the indicator of tumor growth. All animal care procedures were in compliance with institutional guidelines. After one month, lumps were excised, fixed in 4% paraformaldehyde, and paraffin embedded routinely. In addition, cultured 5 × 10^5^ CHH-1 cells were injected into the tail vein of other group of 6-week-old male NOD/SCID mice (*n* = 6). After 40 days, tissues were excised, fixed in 4% paraformaldehyde, paraffin embedded routinely, sliced into 4-μm sections, and stained with hematoxylin and eosin (H&E). Bone marrow of these mice was harvested and treated with red blood cell (RBC) lysis buffer (Sigma-Aldrich). One part of the bone marrow was labeled with monoclonal antibody against human CD45 (BD Biosciences), another part was cytocentrifuged onto slides to stained with Wright.

The research titled “Establishment of a novel human lymphoblastic cell strain with the long arm of chromosome 11 aberration without MLL rearrangement” has obtained human research ethics approval from the Ethics Committee of HuaShan Hospital Affiliated of FuDan University on September 2013. The author has conducted the research as a member of a project or course approved by the Ethics Committee. The original application for ethics approval are filed at the Ethics office, and letter of approval please view the addendum. Inquires may be directed to that office (021-5288-7102).

### Immunohischemistry

Immunohischemistry staining was conducted on the paraffin embedded sections. Tissue sections were deparaffinized in 100% xylene and re-hydrated in descending ethanol series and water according to standard protocols. Heat induced antigen retrieval was performed in citrate buffer and boiled for 10 minutes. After natural cooling, endogenous peroxidase activity was sealed with 3% hydrogen peroxide and non-specific antigen was blocked with 10% goat serum/PBS for 30 minutes at room temperature. Then, the sections were incubated overnight at 4 °C with the following primary antibodies: CD10 (1:50), CD19 (1:200), CD38 (1:200), CD3 (1:30), CD22 (1:200), c-myc (1:200), Bcl-2 (1:100), Bcl-6 (ready to use), CD123 (1:100), and SOX11 (1:200), purchased from Abcam; CD34 (1:400), MPO (1:500), CD5 (1:100), CD138 (1:50), CD56 (1:50), CD13 (1:50), CD1α (1:20), CD4 (1:100), CD8 (1:100), CD23 (1:100), k (1:2500), λ (1:2500), and S100 (1:400), obtained from Dako; CD20 (1:100), CD79α (1:100), and Ki-67 (1:100), obtained from Zymed and TdT (ready to use); CyclinD1 (1:50) obtained from NeoMarkers. After washing with PBS, the sections were reacted with biotinylated anti-IgG secondary antibody (Jackson ImmunoResearch Laboratories, West Grove, PA) for 30 minutes at 37 °C. After washing with PBS for five minutes ×3, the sections were incubated using a VECTA STAIN Elite ABC standard kit (Vector Laboratories) for 30 minutes at 37 °C. Signals were visualized using a diaminobenzidine substrate kit (Vector Laboratories).

### Cell invasion assay

Transwell 24-well plates (Corning) coated with diluted growth factor-reduced Matrigel matrix (BD Biosciences) were used to assess cell invasion. In the upper chamber, 1 × 10^5^ cells were seeded in serum-free RPMI-1640. In the lower chamber, 10% FBS was used as a chemottractant. After 24 hours of incubation, the number of viable cells that migrated into the lower chamber was calculated by trypan blue staining, and cells that adhered on the lower surface of the upper chamber membrane were stained with 0.1% crystal violet for 20 minutes at room temperature. Then, absorbance was detected at 560 nm. Each experiment was replicated four times, and results were calculated over three independent experiments.

### Western blot analysis

Cells were lysed in lysis buffer on ice for 20 minutes. The lysate was centrifuged at 12,000 rpm for 20 minutes at 4 °C. The protein content of the lysate was determined using a protein assay kit (Bio-Rad Laboratories, Hercules, CA, USA). Equivalent amounts of proteins of cell lysates were boiled with 2× sodium dodecyl sulfate sample buffer for five minutes. Proteins were loaded onto polyacrylamide gels and transferred onto polyvinylidine difluoride membranes (Millipore, Bedford, MA, USA). The membranes were blocked by 1% bovine serum albumin PBS Tween 20 and probed with the following primary antibodies for two hours at room temperature: 1:1,000 matrix metalloproteinase-2 (MMP-2) and 1:1,000 MMP-9 (Cell Signaling Technologies Inc.); 1:11,000 human telomerase reverse transcriptase (hTERT, Santa Cruz Biotechnolgy, Santa Cruz, CA); 1:5,000 glyceraldehyde-3-phosphate dehydrogenase (GAPDH, Sigma-Aldrich Corporation, St Louis, Mo, USA). Blots were probed with secondary antibody-conjugated horseradish peroxidase, and were developed using the enhanced chemiluminescence system (Amersham Pharmacia Biotech, Bucks, UK) with enhanced chemiluminescence system film, according to manufacturer’s specifications.

### Measurement of cytokine concentrations and telomerase activity

In order to examine the cytokines secreted by cells, enzyme-linked immunosorbent assay (ELISA) was performed to detect Flt3lig, B cell activating factor (BAFF), IL-2, IL-6, IL-10, LIF, SCF, IL-22, TNF-α, IL-15, IL-4 and IL-7 (Abnova Corporation, Abnova, USA) in the culture supernatant harvested after 72 hours of cultivation without FBS. The quantification of telomerase activity of cells was assayed using the telomerase PCR ELISA kit (Roche Diagnostics GmbH, Mannheim, Germany) based on the telomeric repeat amplification protocol (TRAP).

### Reverse transcriptase polymerase chain reaction (RT-PCR) analysis

Total RNA was isolated from cells using an RNeasy Mini kit (Qiagen, Hilden, Germany), according to manufacturer’s instructions; and incubated with 1 U DNase for 30 minutes at 37 °C to eliminate genomic DNA. Total RNA (100 ng) was reverse-transcribed using a RT kit (GE Healthcare, Tokyo, Japan). All primer sequences are listed in Supplemental Table 1. PCR products were visualized in 1% agarose gels stained with ethidium bromide.

### Statistical analysis

Results are presented as mean ± SD. Statistical differences between multiple groups were compared with one-way ANOVA, and Bonferroni’s multiple comparison was used to determine intergroup quantitative differences. Cytokine response analysis was performed by paired Student’s *t*-test. GraphPad Prism 6.0 (San Diego, CA) was used to analyze the results and create graphs. *P* < 0.05 was considered statistically significant.

## Results

### Establishment of the cell strain

After the 12-week culture of primary bone marrow mononuclear cells, a stable and prominent cell population was observed proliferating gradually, independent of the cytokines. These cells were monoclonally cultured for four weeks, and the culture was continued in RPMI-1640 medium containing 10% FBS for more than 120 passages by serial transfer over two years. The obtained cells were designated as CHH-1 and will be made available. No feeder layer was used at the initiation of the cell culture. The cell strain was adapted to grow in RPMI-1640 medium with 10% FBS alone, with a saturation density of 2 × 10^6^ cells/ml. The cells proliferated consistently as a free-floating single-cell suspension, and were negative for EBV, HBV, HCV, HIV, HTLV-I/II infection, as well as mycoplasma infection, by PCR assay. The cell strain maintained the same properties during more than two years of continuous culture, and sustained its functional characteristics without consequent alteration after freezing and thawing. The cell strain could be frozen under standard conditions using 70% medium, 20% FBS and 10% dimethyl sulphoxide (DMSO), and be revived successfully after storage in liquid nitrogen, with more than 80% viability.

### Morphological and cytochemical characteristics

**Figure 1:**
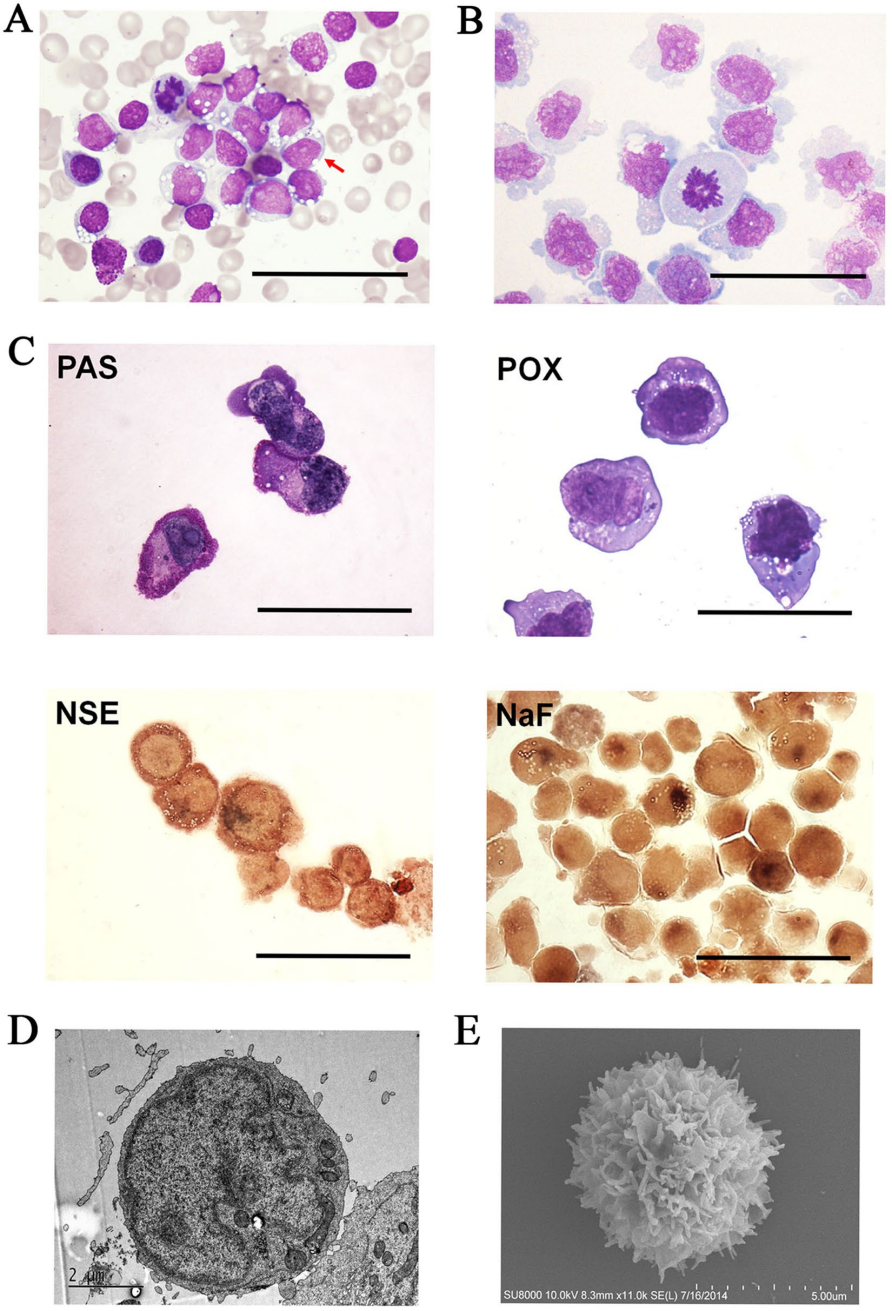
Morphology of CHH-1 cells. Wright’s stains of (**A**) primary leukemia cells in bone marrow of the patient and (**B**) CHH-1 cells. The cells have a blastic appearance with immature nuclei, prominent nucleoli and gray blue cytoplasm with vacuoles, similar to the original leukemia cells. (**C**) Cytochemical staining of CHH-1 cells. (**D**) Ultrastructural appearance of a CHH-1 cell illustrating a deep-folded nucleus with well-defined nucleolus and cytoplasm containing few organelles except a number of mitochondria and rough endoplasmic reticulum. (**E**) Scanning electron microscopy of CHH-1 cells showing abundant microvili. Original magnification ×1000, scale bar 50 μm (**A**,**B**,**C**).

The morphologic appearance of CHH-1 cells resembled that of lymphoblastic leukemia cells in the patient’s bone marrow smear at diagnosis. Wright’s staining revealed that CHH-1 cells were usually round in shape, as well as some cytoplasmic protrusions and basophilic cytoplasm with vacuole formation, similar to primary leukemia cells. The nucleocytoplasmic ratio of these cells was high. Morphologically, the nuclei were irregularly indented or convoluted with one or more prominent nucleoli, and contained finely dispersed chromatin. Mitotic figures were usually observed, and binucleated or multinucleated cells were present (Fig. 1A and B). Cytochemical study revealed that CHH-1 cells were positive for PAS and NSE without being inhibited by NaF treatment, while these were negative for POX (Fig. 1C).

Transmission electron microscopy revealed that most cells had single, large and eccentric nuclei with well-defined nucleoli and a fine chromatin network. Few organelles were present in the cytoplasm, except for some mitochondira and rough endoplasmic reticula (ER) (Fig. 1D). Scanning electron microscopy revealed abundant microvilli on the CHH-1 cell surface (Fig. 1E).

### Immunological marker analysis

The detailed immunophenotypic characterization of the CHH-1 cell strain is summarized in Table 1, along with the comparable data for primary leukemia cells. CHH-1 cells exhibited an immunoprofile typical of B lymphoblastic cell lines: the expression of B-cell associated CD antigens: CD79α, CD19, CD20, CD22 and CD23; the expression of various lymphoblastic markers including CD34, TdT, HLA-DR and CD10; the lack of myeloid-, T-, NK- and plasmocyte-associated markers; the negative cyIgM and positive CD38 expression in early-stage B cells. The immunophenotype was compatible with that of the common B-ALL subgroup, according to EGIL classification.

**Table 1:** Immunophenotypic marker profiles of the original patient leukemia cells, CHH-1 cells and the tumor formed by CHH-1 cells.

	Patient	CHH-1	Tumor		Patient	CHH-1	Tumor
CD34	79.93%	53.40%	−	CD13	−	−	−
TdT	89.12%	98.80%	+	CD14	−	−	N
HLA-DR	90.60%	100.00%	N	CD15	−	−	N
CD10	87.16%	67.50%	−	CD1α	−	−	−
CD19	87.35%	81.80%	+	CD16	−	−	N
CD79α	90.10%	99.00%	+	CD4	−	N	−
CD20	67.36%	94.40%	+	CD8	−	N	−
CD38	86.00%	99.80%	+	CD22	N	99.60%	+
cyIgM	−	−	N	CD23	N	92.40%	+
CD3	−	−	−	Kappa	N	−	−
CD2	−	−	N	Lammda	N	−	−
CD5	−	−	−	c-myc	N	N	+
CD7	−	−	N	Bcl-2	N	N	+
CD138	−	−	−	Bcl-6	N	N	−
CD56	−	−	+	CD123	N	N	−
CD64	−	−	N	CyclinD1	N	N	−
MPO	−	−	−	S100	N	N	−
CD117	−	−	N	SOX11	N	N	−
CD33	−	−	N	Ki-67	N	N	>90%
CD11c	−	−	N				

For the original patient leukemia cells and CHH-1 cells, the values shown are the means of percent positive cells in three tests analyzed by flow cytometry. For the tumor formed by CHH-1 cells, the qualitative results are detected by immunohistochemistry. CD: cluster of differentiation; MPO: myeloperoxidase; TdT: terminal deoxynucleotidyl transferase; HLA: human leukocyte antigen; cy: cytoplasmic expression; N: not tested.

### Cytogenetics

**Figure 2:**
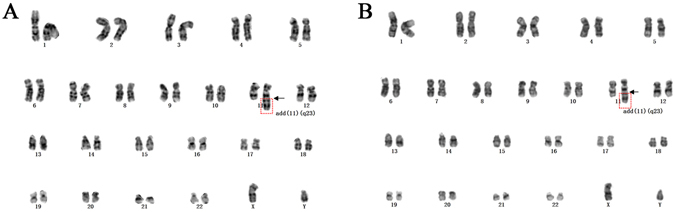
Cytogene analysis. G-banding karyotype of (**A**) leukemia cells of the patient and (**B**) CHH-1 cells. The cells show the same structural abnormality in the long arm of chromosome 11 which is indicated by arrows as the primary leukemia cells.

Chromosome analysis was carried out using the G-banding technique, showing that the CHH-1 cell strain had structural abnormalities consistent with the blasts originally recovered from the patient. Complete karyotyping of CHH-1 cells after the 2-month culture revealed that all cells revealed 46, XY, add(11)(q23) (Fig. 2). After the 18-month culture, the karyotype of CHH-1 cells remained stable. FISH using a probe specific for *MLL* failed to detect its rearrangement or amplification in CHH-1 cells, as was the case with primary leukemia cells (Supplemental Fig. 1A). This result also matched the molecular test performed at diagnosis, in which no chimaeric mRNAs associated with *MLL* rearrangement were detected in the patient’s bone marrow cells; suggesting that the breakpoint in the long arm of chromosome 11 did not affect the *MLL* gene, and that the breakpoint site may lie below the location of the *MLL* gene near the telomere region.

Ig gene rearrangement analysis revealed that CHH-1 cells and leukemia cells derived from the patient contained the same rearranged Ig heavy chain (*Ig*-*VH* and *Ig*-*DH*) and *Ig*-*κ* genes (Supplemental Fig. 1B). These cytogenetic and Ig gene rearrangement analyses documented that cell strain CHH-1 was clonally derived from the patient’s original leukemia cells.

### Proliferation and clonal growth in semisolid medium

CHH-1 cells stably proliferated in RPMI-1640 medium containing 10% FBS with a population doubling time of 36 hours. The cell cycle analysis of different cell population doubling levels (PDLs) of CHH-1 cells revealed that the percentages of G0/G1, G2/M and S phase cells were similar without statistical difference (*P* > 0.05), and a greater proportion of cells was in the G2/M or S phase (Supplemental Fig. 2A), suggesting that CHH-1 cells could maintain the cell division cycle stably during continuous culture *in vitro*, and a great proportion of cells may be in the proliferation and division state.

Knowing that the colony-forming capacity in the semisolid medium reflects the proliferative capacity of a single cancer cell, we estimated the colony-forming capacity of different PDLs in CHH-1 cells in 1% methylcellulose semisolid medium. Colonies were observed six days after a single cell was cultured in the semisolid medium (Supplemental Fig. 2B). The colony-forming efficiency of 60PDL, 120PDL and 210PDL in CHH-1 cells was 50.83 ± 3.25%, 49.00 ± 2.64% and 52.67 ± 3.21%, respectively, which did not reveal any statistically significant difference (*P* > 0.05) after the 14-day culture; while normal human CD19+ PBMCs were unable to form colonies. These results suggest that the colony-forming capability of CHH-1 cells was high and could maintain its stability during continuous culture.

### Tumorigenicity in NOD/SCID mice

**Figure 3:**
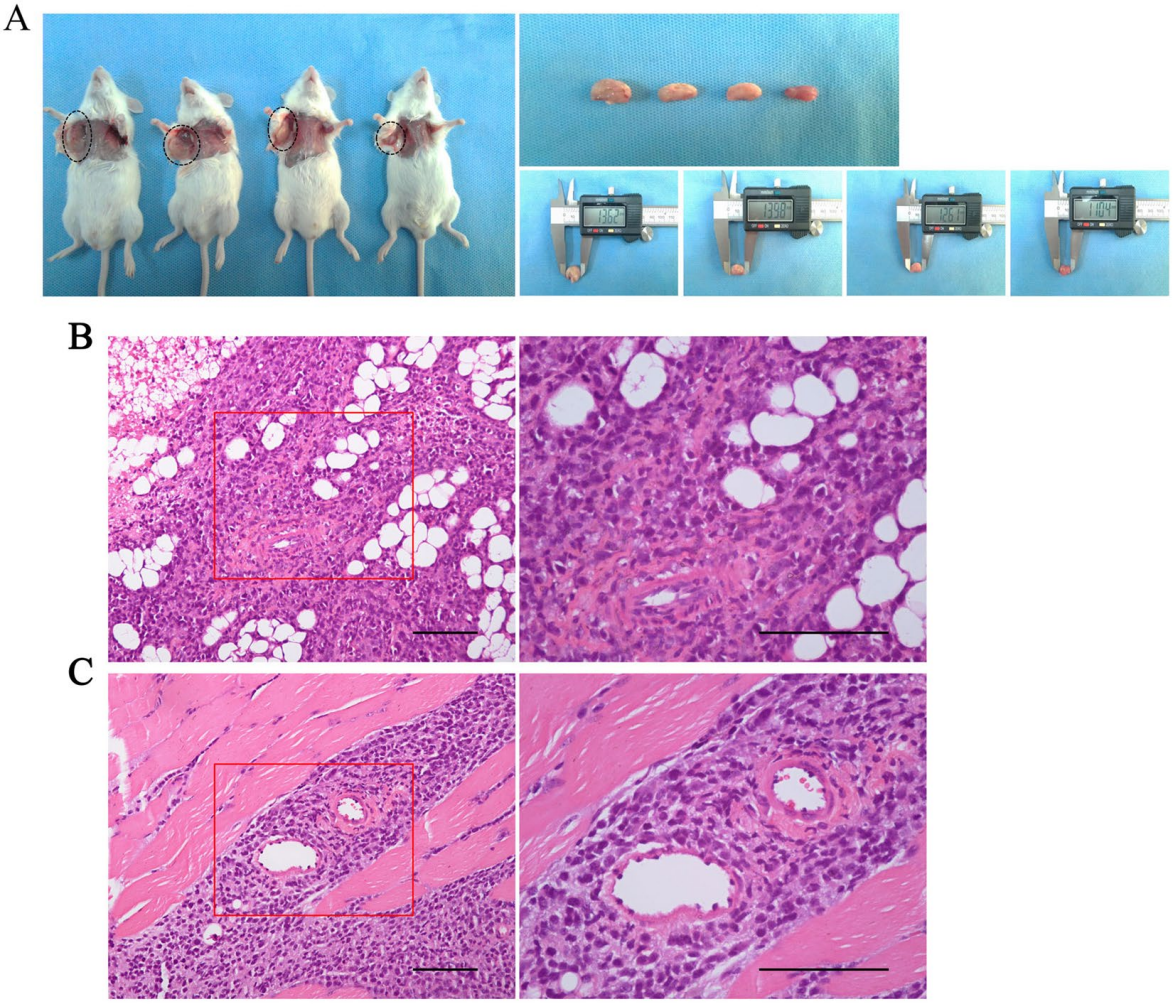
Tumorigenicity of CHH-1 cells. (**A**) A representative subcutaneous tumor is observed (circle indicated) in the right armpit of NOD/SCID mouse after subcutaneous injection of 5 × 10^6^ CHH-1 cells (*n* = 12, the results are representative of three separate experiments). H&E staining of the tumor notes CHH-1 cells infiltrating the (**B**) adipose tissue and (**C**) striated muscle diffusely. The right panel is a magnification of the outlined area in the left panel. Original magnification: left panel ×200, right panel ×400, scale bar 100 μm.

In order to determine the tumorigenicity of CHH-1 cells, 5 × 10^6^ cells were injected subcutaneously into NOD/SCID mice. After 28 days, subcutaneous tumors were observed in all mice at a mean volume of 663.19 ± 320.85 mm^3^ (Fig. 3A). Histological examination uncovered that malignant lymphoblastic cells proliferated and infiltrated the subcutaneous adipose and muscle tissues diffusely (Fig. 3B and C). Immunohistochemical analysis revealed that the infiltrated cells hybridized by anti-human antibodies were positive for B lymphoblastic cell markers TdT, CD79α, CD20, CD19, CD22 and CD23, and negative for myeloid-, T-, NK-, plasmocyte-, Langerhans cell-, and other mature B lymphoma subtype-associated markers. The overexpression of oncoprotein c-myc and anti-apoptotic protein Bcl-2, which are known as poor prognosis factors^10, 11^, was also detected. Remarkably, the percentage of nuclear Ki-67 positive cells was over 90%, indicating that most cells were in proliferation. Results of the immunophenotype comparison between primary leukemia cells, CHH-1 cells and tumors formed by CHH-1 cells are listed in Table 1.

Additionally, suspension cells separated from tumors could also be monoclonally cultured and passaged continuously *in vitro*. The morphological characteristics of the suspension cells were similar to CHH-1 cells. The karyotype of the monoclonal suspension cells was also 46, XY, add(11)(q23), and the *MLL* rearrangement was not detected by FISH, which was the case with CHH-1 cells. In addition, the Ig gene rearrangement of monoclonal suspension cells was also the same as CHH-1 cells. These findings confirmed that the malignant lymphoblastic cells in the tumor were derived from CHH-1 cells and maintained its karyotype abnormality during the neoplastic processes.

### Invasion

**Figure 4:**
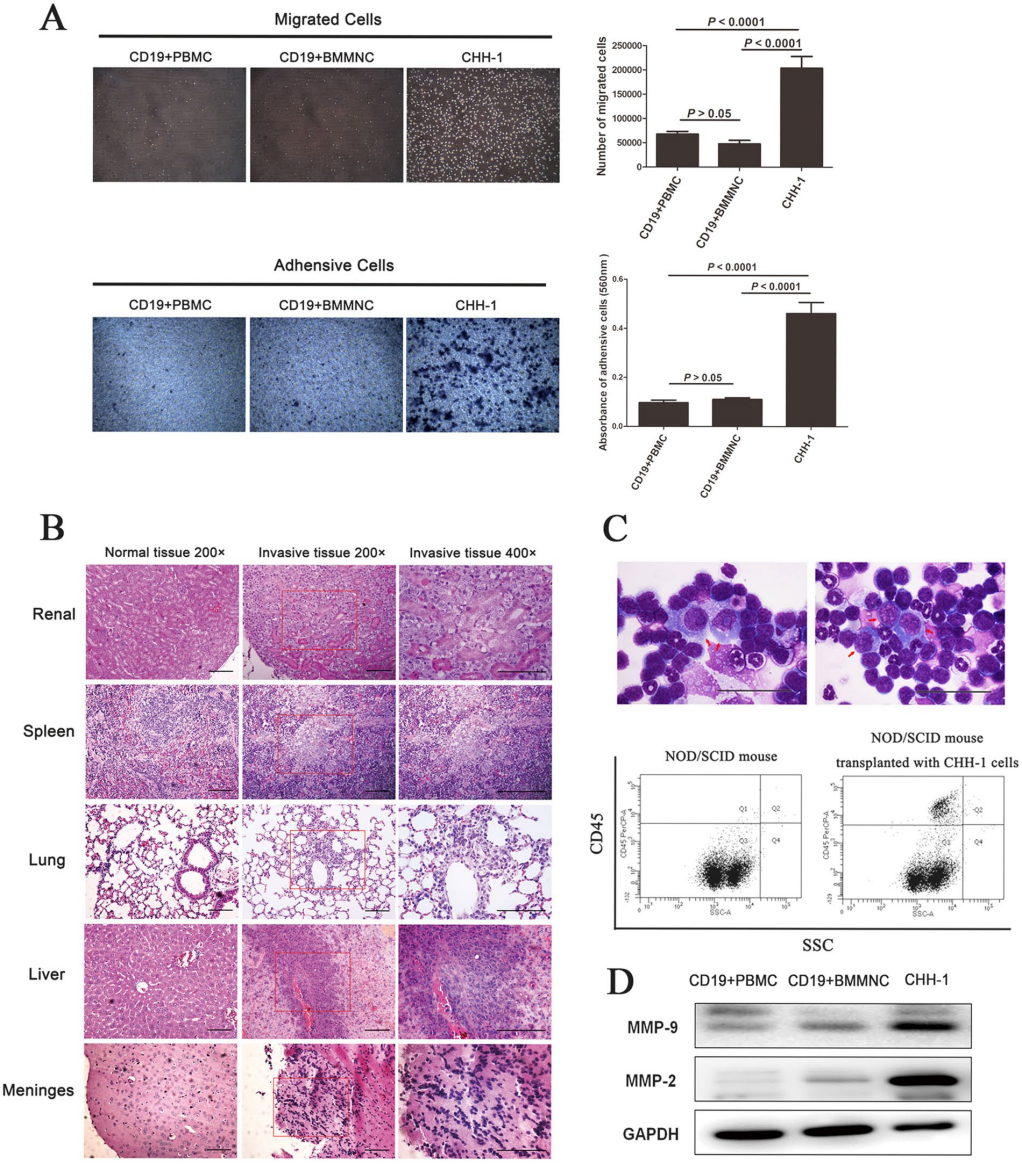
Invasive ability of CHH-1 cells. (**A**) The number of CHH-1 cells passing through or adhering on the transwell chambers coated with Matrigel is significantly greater than that of the control cells. Data represent the mean ± SD of three independent experiments. CD19+ PBMCs and CD19+ BMMNCs derived from the normal human are used as control cells. (**B**) Histopathology of CHH-1 cells invading in the kidney, spleen, lung, liver and meninges of NOD/SCID mice is observed 40 days after injection of 5 × 10^5^ cells into the tail vein (*n* = 6, the results are representative of two separate experiments). (**C**) CHH-1 cells with representatively atypical morphology are observed (arrow indicated) in the NOD/SCID mice bone marrow centrifuged smears, and the engraftment presented as human CD45+ cells (Q1 + Q2) in the mice bone marrow is detected by flow cytometry 40 days after transplantation via tail vein (*n* = 6). (**D**) Overexpression of MMP-9 and MMP-2 in CHH-1 cells is assayed by Western blot. GAPDH is used as an internal control. Original magnification: ×200, ×400, scale bar 100 μm (**A**,**B**), ×1000, scale bar 50 μm (**C**).

Knowing that invasion is one of the malignant features of ALL, we investigated the invasive ability of CHH-1 cells. Transwell coated with Matrigel *in vitro* revealed that the number of CHH-1 cells migrating through the Matrigel was significantly greater than that of normal human CD19+ PBMCs and CD19+ BMMNCs (Fig. 4A). Nearly all CHH-1 cells migrated very efficiently across the Matrigel-containing chambers. Subsequently, we injected 5 × 10^5^ cells into the tail vein of NOD/SCID mice, and observed the diffuse infiltration of CHH-1 cells in the kidneys, spleen, lungs, liver and meninges of mice 40 days later (Fig. 4B). In addition, CHH-1 cells were also observed in these mice bone marrow smears, and the percentage of CHH-1 cells which positive for human monoclonal antibody CD45 was 9.41 ± 2.52% detected in the mice bone marrow by flow cytometry (Fig. 4C). These results show that CHH-1 cells were highly invasive and consistent with the overexpression of MMP-2 and MMP-9, which are two of the most important proteins that promote cancer cell invasion^12, 13^. This was detected in the cell strain by western blot (Fig. 4D).

### Telomerase activity and cytokines

**Figure 5:**
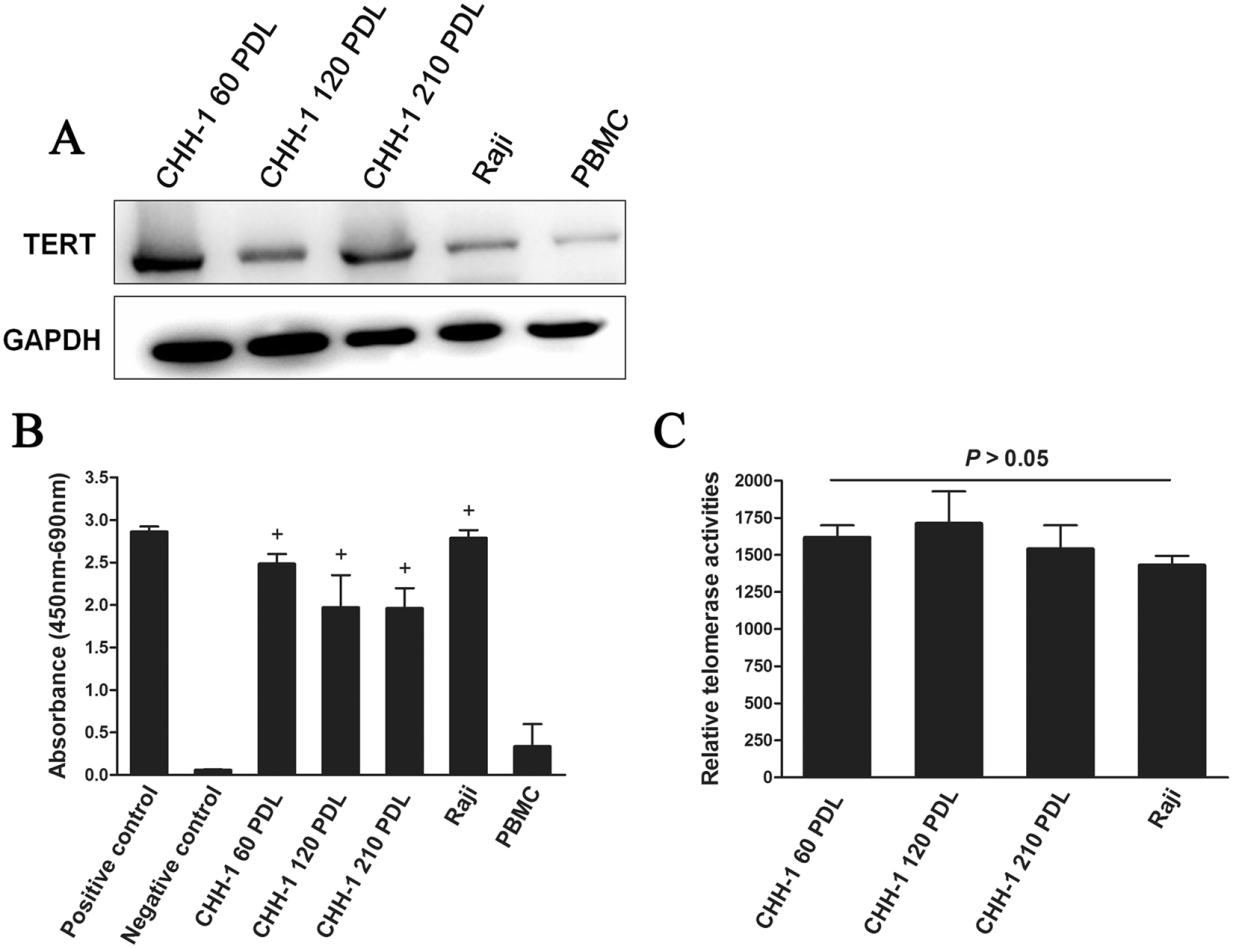
Telomerase activity of CHH-1 cells. (**A**) TERT expression can be detected expression in different PDLs of CHH-1 cells by Western blot. GAPDH is used as an internal control. TRAP-ELISA analysis demonstrates that (**B**) the telomerase of CHH-1 cells is activated, and (**C**) the relative telomerase activity of different PDLs CHH-1 cells is insignificantly high as compared with Raji cells. ^+^Means telomerase of cells is activated. Raji cell line is used as a positive control. PBMCs from a normal person are used as a negative control. Values are given as mean ± SD of three independent experiments.

Due to the “end replication problem”, telomeres undergo shortening with cell division that acts as a mitotic clock, and triggers entry into senescence^14^. The maintenance of telomeres contributes to the immortal phenotype. Since telomerase activation contributes to the maintenance of telomere length, constitutive telomerase activation in cancer cells may account for the immortality of tumor cells^15, 16^. In the CHH-1 cell strain, we found that both hTERT and telomerase were overexpressed (Fig. 5A and B), and that relative telomerase activity remained high in different PDL cells (Fig. 5C). These results demonstrate the unlimited division capability of CHH-1, and further confirm that CHH-1 is an immortal cell strain.

**Figure 6:**
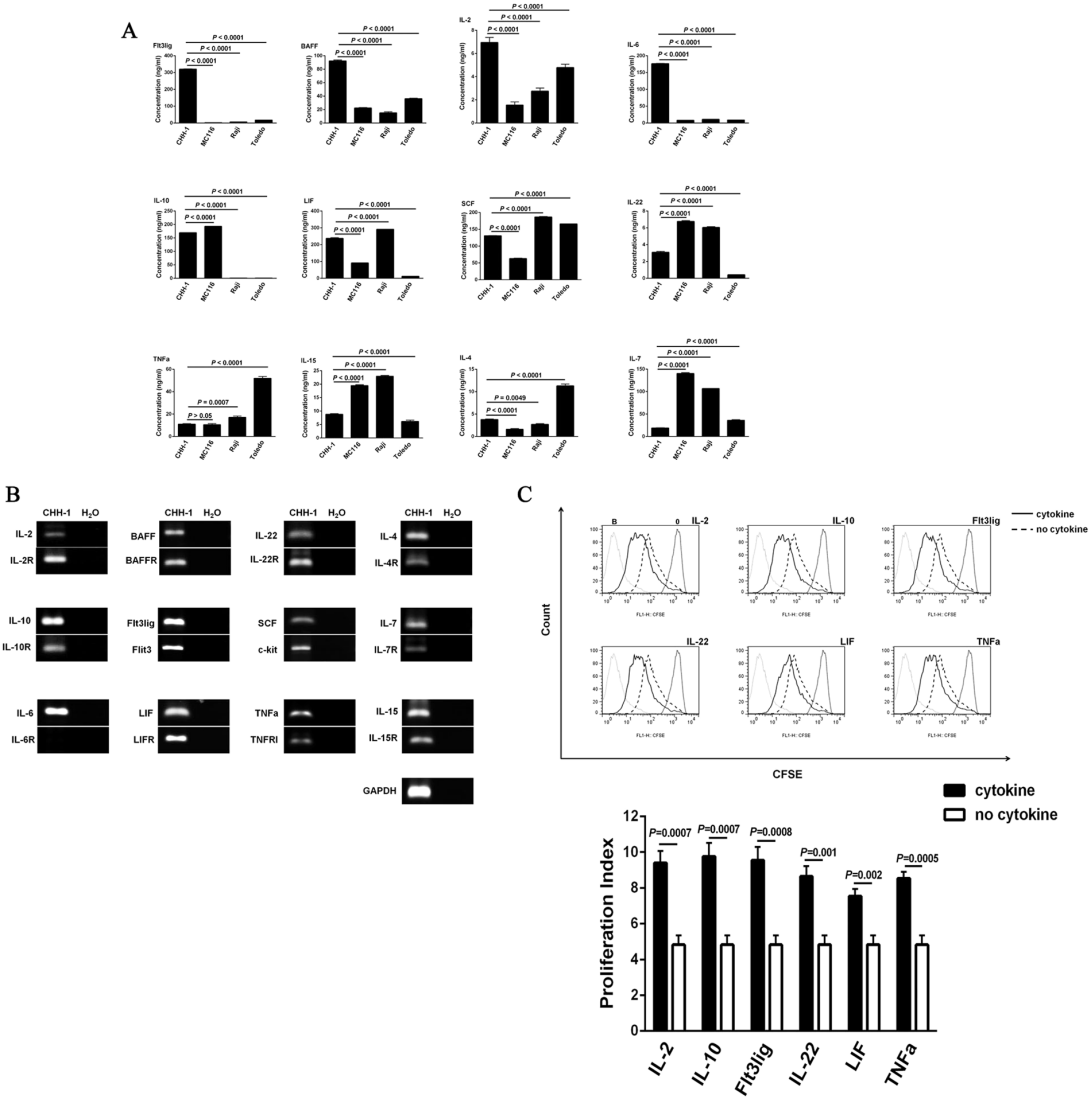
Cytokines secreted by CHH-1 cells and their proliferative effect on the cells. (**A**) The concentration of different cytokines in the culture supernatant secreted by CHH-1 cells as compared with MC116, Raji and Toledo cells as detected by ELISA. (**B**) Transcription of the secreted cytokines and their corresponding receptors is detected in CHH-1 cells by RT-PCR. GAPDH is used as an internal control. (**C**) The effect of the cytokines on CHH-1 cell proliferation is determined by CFDA-SE assay. Values represent the mean ± SD of three independent experiments.

In addition, we examined the soluble cytokines in the culture supernatant secreted by CHH-1 cells, and compared these with other leukemia-lymphoma cells such as MC116, Raji and Toledo. Cytokines such as human Flt3lig, BAFF, IL-2, IL-6, IL-10, LIF, SCF, IL-22, TNFα, IL-15, IL-4 and IL-7 were detectable in the culture supernatants of these cells by ELISA. In CHH-1 cells, the secretion contents of Flt3lig, BAFF, IL-2 and IL-6 were remarkably higher than those in the other cell lines. In addition, the secretion contents of IL-10, LIF and SCF were also abundant in CHH-1 cells (Fig. 6A). RT-PCR detected the transcripts of IL-2, BAFF, IL-22, IL-4, IL-10, Flt3lig, SCF, IL-7, LIF, TNFα, IL-15 and the corresponding receptors expressed in CHH-1 cells; which means that these cytokines may be autocrined by CHH-1 cells (Fig. 6B). Furthermore, we examined the response of CHH-1 cells to some cytokines that may be autocrined, including IL-2, IL-10, Flt3lig, IL-22, LIF and TNFα. As shown in Fig. 6C, all these cytokines can induce a significant proliferation of CHH-1 cells. These results suggest that CHH-1 cells had a unique growth factor autocrine mode that may promote self-proliferation.

## Discussion

Structural abnormalities in the long arm of chromosome 11 frequently occur in various hematologic neoplasias. Particularly, chromosome band 11q23 is relatively a common cytogenetic alteration in adult ALL^17^. Furthermore, most present studies have focused on 11q23 translocation that disrupts the *MLL* gene, known as the prognostic biomarker of high-risk disease^18^. However, due to the marked heterogeneity of rearrangements in this chromosomal region, the presence of 11q23 abnormalities does not always correlate with *MLL* rearrangement^19, 20^. In fact, 11q23 abnormalities without *MLL* involvement have been reported in ALL, acute myeloid leukemia (AML), myelodysplastic syndrome (MDS) and malignant lymphomas^7, 21–26^. However, there is little knowledge about this type of chromosome 11 aberration. One of the most important factors is the lack of corresponding cell models. Among the various abnormalities in the 11q23 region, add(11)(q23) chromosome structure aberration has been reported in multiple malignant hematologies^7^. CHH-1 is the cell strain derived from a B-ALL patient carrying add(11)(q23) chromosome structure aberration without *MLL* rearrangement or other chromosomal abnormalities. The cell strain can maintain the same genetic abnormality stably during continuous passaging *in vitro*. Knowing that cells that can be monoclonally cultured and derived from tumors formed by CHH-1 cells also contain the same cytogenetic aberration, we speculate that the tumorigenicity of CHH-1 cells may be associated with this cytogenetic abnormality. Except *MLL*, there are many other proto-oncogenes and tumor suppressor genes such as *ETS1* ^27^, *FLI1* ^28^, *LARG* ^29^, *CADM1* ^30^, *DDX6* ^31^, *PLZF* ^32^, *TSG11* and *THY1* that are located between 11q23 and 11q25. In addition, the break in and the additional material on the long arm of chromosome 11 may create various genetic aberrations including new translocation, amplification and deletion. These unidentified genetic aberrations in the long arm of chromosome 11 other than *MLL* may provide important insights into the molecular genetic mechanism underlying the pathogenesis of hematopoietic malignancies. Although the structural abnormality of add(11)(q23) in different cases may not be all concordant, the CHH-1 cell strain can provide a novel and stable model to explore the function of other proto-oncogenes or tumor suppressors and new genetic aberrations in chromosome 11 in the initiation and maintenance of malignant clones.

As the CHH-1 cell strain in our study was obtained by monoclonal culture, it was composed of homogeneous blastic cells with differentiation arrest. As authenticated by the morphological, immunologic, cytochemical and Ig isotypic characteristics of the cell strain, CHH-1 cells are the same clone derived from the patient’s primary leukemia cells. The high colony-forming capacity in semisolid medium, tumorigenicity in NOD/SCID mice and high invasion ability reflect the neoplastic nature of this cell strain. Until now, the CHH-1 cell strain has been passaged continuously over two years (over 200 PDL) *in vitro* and has continuously maintained its stability of proliferation and cytogenetical feature. Therefore, this cell strain can represent corresponding human leukemia cells in patients and be used as a permanently stable cell model in the research of ALL or lymphoblastic lymphoma.

In addition, the CHH-1 cell strain also exhibited the characteristics of the unique autocrine mode and high telomerase activity consistent with the behavior of hematological malignances. Cytokines are essential for the “leukemic” microenviroment by fine tuning cross talks between leukemia cells through a myriad of different signaling pathways to support their persistence^33^. A study on B-ALL patients discovered that the expression levels of cytokines IL-7, IL-10 and IL-15 and their receptors were higher than those in healthy controls, suggesting the existence of a complex autocrine/paracrine mechanism underlying the regulation of leukemia cell functions^34^. It was found in the present study that CHH-1 cells could secrete multiple soluble growth factors, co-express corresponding receptors and ligands at the transcriptional level, and proliferate in response to such cytokines; implying the possibility of autocrine stimulatory loops in the cell strain. Compared with other leukemia-lymphoma cell lines, the CHH-1 cell strain represents a unique autocrine model. For instance, the high concentration of Flt3lig secreted by the strain can stimulate the proliferation of CHH-1 cells *via* Flt3 co-expression in the same cells. Flt3lig, known as the cognate ligand for Flt3, has been reported to promote the survival of primitive hematopoietic progenitor cells with B-cell potential^35^. Flt3lig and its receptor (Flt3) also contribute to the pathogenesis of leukemia. The cell surface expression of Flt3 was observed in approximately 64% of B-ALL cases, suggesting that this specific ligand-receptor interaction could either induce cell proliferation or prevent cell apoptosis^36^. However, the detailed mechanism of this key hematopoietic regulatory ligand-receptor system of Flt3lig-Flt3 in leukemogenesis remains to be investigated. CHH-1 cell strain could be used in the field of some specific microenvironmental regulation in leukemogenesis such as this. The reactivation of telomerase is known as the key hallmark of cancer, allowing replicative immortality^15, 37, 38^ and stabilizing the mutant genome by evading cell senescence^39, 40^. The elevation of telomerase activity was observed in approximately 75% of patients with acute leukemia^41, 42^. Moreover, recent studies have demonstrated that the high level of telomerase activity was associated with significant poorer prognosis^42^ or indicated the relapse phase in patients with acute leukemia, while it decreases to normal level when CR was achieved^43^. These findings imply that this telomerase activity could be used to detect residual disease and monitor the disease condition in hematological malignancies. In recent years, anti-telomerase therapy has also been developed in the treatment of hematologic neplasia^44^. Hence, the CHH-1 cell strain can be utilized in such relevant studies. In addition, due to the overexpression of *c*-*myc*, which is known as a strong regulator of telomerase^45, 46^ and chromosome aberration, this cell strain could also be used to explore the interaction of telomerase activity and other oncogenic factors in the malignant process.

In conclusion, CHH-1 is a stable and permanent human B-lymphoblastic cell line with add(11)(q23) chromosome structural aberration. This novel hematological malignant cell strain may provide a favorable cell model for the research of human leukemias. The distinctive genetic abnormality and features of CHH-1 could help identify the unexplored territory of similar chromosome 11 structural aberrations in leukemia, and provide new insight into the pathogenesis, progression, diagnosis, prognosis and target therapy of ALL with such genetic abnormality.

## Electronic supplementary material

Establishment of a novel human lymphoblastic cell strain with the long arm of chromosome 11 aberration without MLL rearrangement
