# Supplementary material for: Establishment of a novel human lymphoblastic cell strain with the long arm of chromosome 11 aberration without MLL rearrangement
Source: Sci Rep. 2017 Apr 13;7:867. doi: 10.1038/s41598-017-00874-6 (PMC5429836; doi:10.1038/s41598-017-00874-6)

**Establishment of a novel human lymphoblastic cell strain with the long arm of chromosome 11 aberration without *MLL* rearrangement**

Qian Wang^1^, Lin Zhuang^1^, Pei Li^1^, Qiang Niu^1^, Ping Zhu^1^, Miao-Xia He^2^, Hui Jiang^2^, Chang-Cheng Liu^3^, Min-Jun Wang^3^, Li Chen^4^, Hui Cheng^4^, Yan Ma^1^, Xiao-Xia Hu^4^, Yi-Ping Hu^3*^, Xiao-Ping Xu^1*^

Supplemental Table 1. Sequences of the primers for RT-PCR

| IL-2 | 5’-GCAACTCCTGTCTTGCATTG-3’ |
| --- | --- |
|  | 5’-ATGTGAGCATCCTGGTGAGT-3’ |
| IL-2R | 5’-GTGGTGGGGCAGATGGTTTA-3’ |
|  | 5’-TTGTGACGAGGCAGGAAGTC-3’ |
| IL-10 | 5’-GAGATGCCTTCAGCAGAGTGA-3’ |
|  | 5’-CCTTGATGTCTGGGTCTTGGT-3’ |
| IL-10R | 5’-ATTCGGGAGATGCCTGGTTG-3’ |
|  | 5’-CATTCCTCAGTGGGCTGGTT-3’ |
| IL-6 | 5’- AATGAGGAGACTTGCCTGGTG-3’ |
|  | 5’-TGGCATTTGTGGTTGGGTCA-3’ |
| IL-6R | 5’-CACGCCTTGGACAGAATCCAG-3’ |
|  | 5’-TGGGCAGTGGTACTGAAGAAGA-3’ |
| BAFF | 5’-CCAGAAGAAACAGGATCTTACACA-3’ |
|  | 5’-CATCCCCAAAGACATGGACCT-3’ |
| BAFFR | 5’-TCATCATTCTGTCTCCGGGA-3’ |
|  | 5’-TCTTGGTGGTCACCAGTTCAG-3’ |
| Flt3lig | 5’-CCGTGAGCTGTCTGACTACC-3’ |
|  | 5’-GTCTGGACGAAGCGAAGACA-3’ |
| Flt3 | 5’-AAACCTCAAGTGCTCGCAGA-3’ |
|  | 5’-GAGTACTGCTCGACACCCAC-3’ |
| LIF | 5’-GTCTTGGCGGCAGTACACA-3’ |
|  | 5’-GGGAGGTGCCAAGGTACAC-3’ |
| LIFR | 5’-GGCTCATCACCACCTTCCAA-3’ |
|  | 5’-ATAAGGCATGGTTCCGACCG-3’ |
| IL-22 | 5’-CCTTCTCTTGGCCCTCTTGG-3’ |
|  | 5’-ATGAAGGTGCGGTTGGTGAT-3’ |
| IL-22R | 5’-CACCTCCCAACTCCCTGAAC-3’ |
|  | 5’-AGGTGATCTCGGACAGGCTA-3’ |
| SCF | 5’-AGCCAGCTCCCTTAGGAATGA-3’ |
|  | 5’-TGCCCTTGTAAGACTTGGCTG-3’ |
| c-kit | 5’-ATTCAAGCACAATGGCACGG-3’ |
|  | 5’-GTGTGGGGATGGATTTGCTC-3’ |
| TNF-α | 5’-TGCACTTTGGAGTGATCGGC-3’ |
|  | 5’-ACTCGGGGTTCGAGAAGATG-3’ |
| TNFRI | 5’-GGAAGAACCAGTACCGGCAT-3’ |
|  | 5’-TCTGGGGTAGGCACAACTTC-3’ |
| IL-4 | 5’-CTTTGCTGCCTCCAAGAACAC-3’ |
|  | 5’-TTCCTGTCGAGCCGTTTCAG-3’ |
| IL-4R | 5’-TGGAGTGTGAGGAGGAGGAG-3’ |
|  | 5’-CCGAAGGTGGAAGAAGGCAT-3’ |
| IL-7 | 5’-CCTTGTTCTGTTGCCAGTAGC-3’ |
|  | 5’-CATACCTTCCTTATTAGCATCACAG-3’ |
| IL-7R | 5’-CTGCCCATCTGAGGATGTAGTC-3’ |
|  | 5’-TTGTAGTCCCAAGGCTAAGCAG-3’ |
| IL-15 | 5’-AACAGAAGCCAACTGGGTGAA-3’ |
|  | 5’-GCATCTCCGGACTCAAGTGAA-3’ |
| IL-15R | 5’-CCCAGCTCAAACAACACAGC-3’ |
|  | 5’-CAGTGGACGTGGAGATAGCC-3’ |
| CD34 | 5’-CTACAACACCTAGTACCCTTGGA-3’ |
|  | 5’-GGTGAACACTGTGCTGATTACA-3’ |
| CD99 | 5’-GAAAGAAGGGGAAGAGGCCG-3’ |
|  | 5’-GCTCTCCATGTCCACCTCC-3’ |
| TdT | 5’-CTCCCGGCAGTTTGAGAGAG-3’ |
|  | 5’-CCAATCCCAGATGCGCAAAA-3’ |
| GAPDH | 5’-GGTGGTCTCCTCTGACTTCAACA-3’ |
|  | 5’-GTTGCTGTAGCCAAATTCGTTGT-3’ |

Sup Fig 1. (A) *MLL* rearrangement is not detected in the primary leukemia cells and CHH-1 cells by FISH analysis using dual-color break-apart *MLL* probe. (B) CHH-1 cells and the primary leukemia cells are confirmed as the same Ig rearrangement by PCR assay.


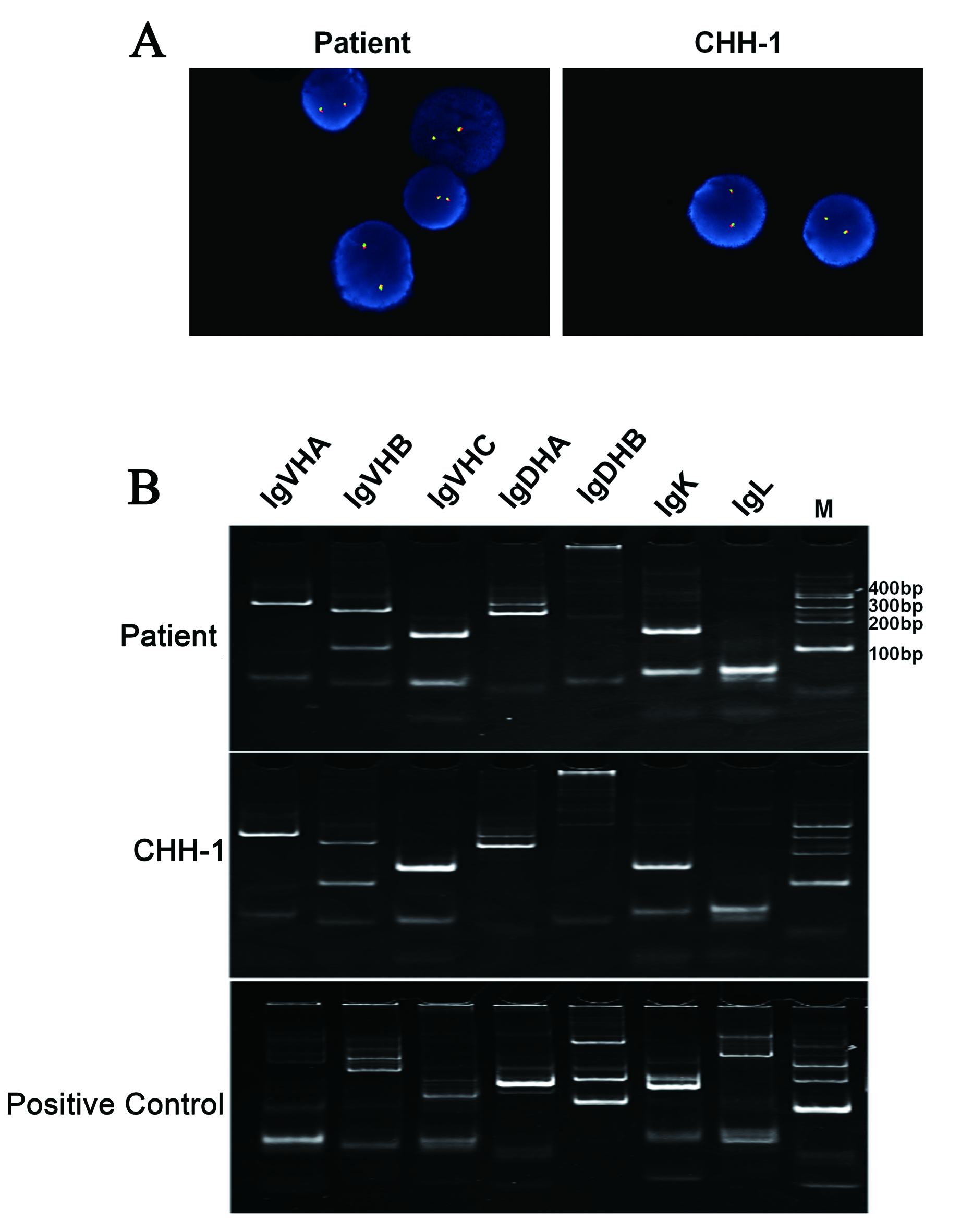


Sup Fig 2. Cell cycle analysis and colony-forming capacity in semisolid medium of CHH-1 cells. (A) Cell cycle distribution of different PDLs CHH-1 cells is analyzed by flow cytometry with PI staining (*n* = 3). (B) The single CHH-1 cell can form colonies after 6-day culture in 1% methylcellulose semisolid medium. Original magnification ×200, scale bar 100 μm.


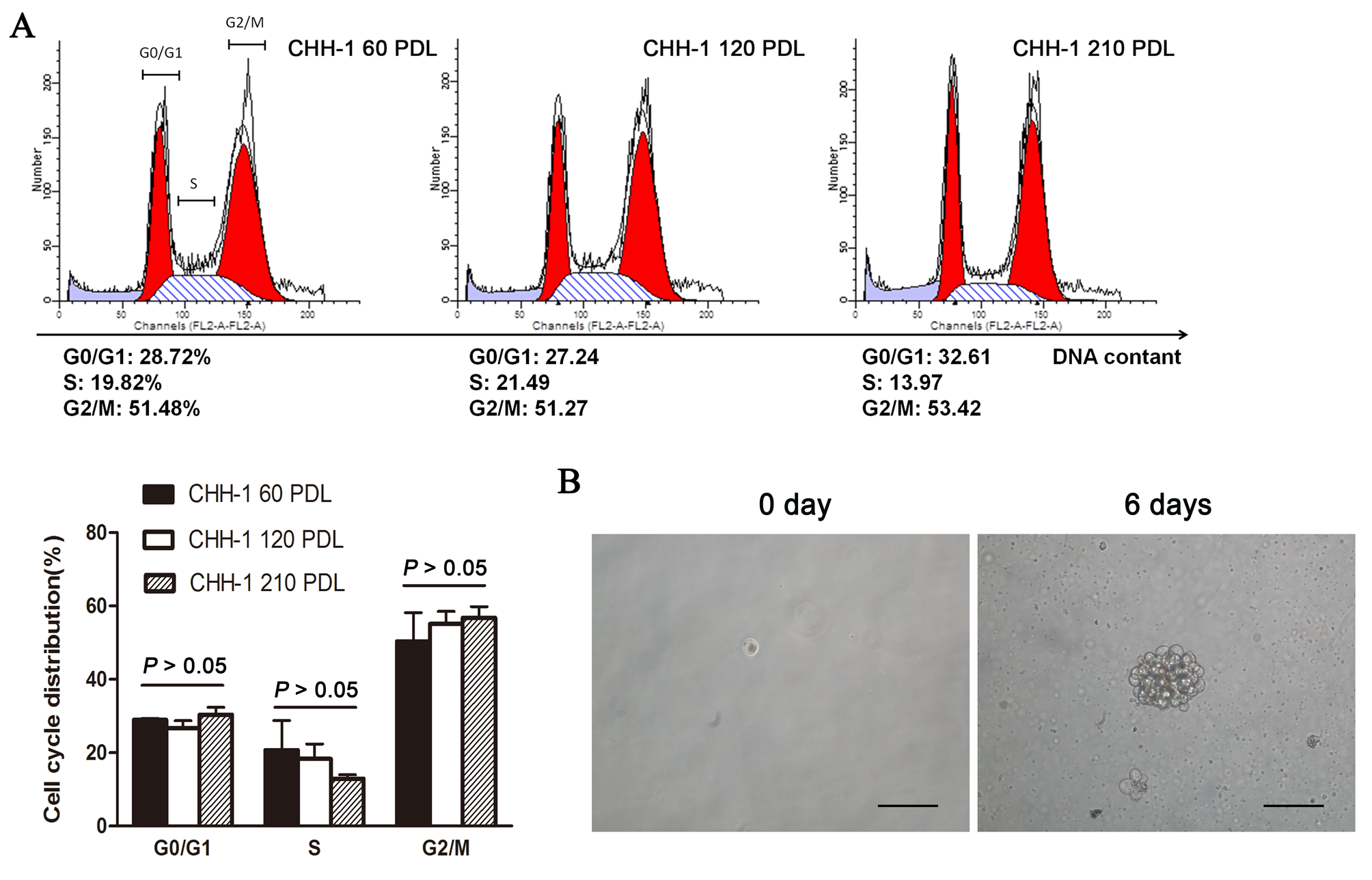

Supplement: Supplementary file 1 — Establishment of a novel human lymphoblastic cell strain with the long arm of chromosome 11 aberration without MLL rearrangement [file 41598_2017_874_MOESM1_ESM.doc]
